# Key Role of Multidisciplinary Collaboration towards Global Elimination of HCV Infection

**DOI:** 10.3390/ijerph19074158

**Published:** 2022-03-31

**Authors:** Laura Krekulova, Zbynek Oktabec, Lee W. Riley

**Affiliations:** 1 4th Department of Internal Medicine, First Faculty of Medicine, Charles University in Prague, Kateřinská 32, 128 00 Prague 2, Czech Republic; krekulova@remedis.cz; 2 Remedis, s.r.o., Vladimírova 10, 140 00 Prague 4, Czech Republic; 3 Department of Addictology, First Faculty of Medicine, Charles University in Prague, Kateřinská 32, 128 00 Prague 2, Czech Republic; 4 Division of Infectious Diseases and Vaccinology, School of Public Health, University of California, Berkeley, CA 94720, USA; lwriley@berkeley.edu

**Keywords:** hepatitis C virus, HCV, HCV infection, viral hepatitis C, people who inject drugs, PWID, global HCV elimination plan, WHO

## Abstract

The elimination of HCV (hepatitis C virus) infection is, according to WHO (World Health Organization), of international interest. With new diagnostic tools and treatment possibilities, one major challenge for the elimination is to involve infected patients, especially those from socially excluded subpopulations, into HCV infection-treatment programs. The key question is how to help people who inject drugs (PWID) to engage in HCV infection-treatment programs and improve communication between PWID and hepatologists or other medical professionals involved in the treatment of chronic HCV infection. Furthermore, the medical professionals have to accept the changing spectrum of patients with chronic viral hepatitis. Without close interdisciplinary cooperation, it would be extremely difficult to achieve the WHO goal of global viral hepatitis C elimination. Here, we try to encourage our colleagues as well as addictologists and social workers to play their crucial part in the viral hepatitis C eradication process. It is extremely important for the healthcare providers to be able to communicate with addicted clients, inform PWID about the latest developments in the diagnosis and HCV infection treatment, and get them motivated to engage with specialized treatment programs.

## 1. Introduction

The pandemic of HCV infection has been the main focus of WHO attention for several years.

This disease is of growing global concern due to its substantial effect on morbidity and mortality [1]. The spectrum of infected patients has changed over the years:

Viral hepatitis C was initially mainly an iatrogenic infectious disease (ID), transmitted by blood and blood products, non-disposable material for injections, etc. Nowadays, however, especially in developed European countries and in the USA, it is primarily an ID linked with people who inject drugs (PWID) due to their risk behaviour [2,3].

With the introduction of new direct-acting antivirals (DAA) in the treatment, our approach to the diagnosis and therapy of this disease has changed dramatically. Nowadays, we are able to cure HCV infection.

The 69th World Health Assembly endorsed the Global Health Sector Strategy for Viral Hepatitis, including the goal to eliminate HCV infection as a public health threat by 2030 [4,5]. WHO introduced global targets for the care and management of viral hepatitis C [5] and emphasised the necessity of involving socially excluded subpopulations (for example PWID, homeless people, prisoners, etc.) in preventive and treatment programs. Beside the financial aspect of diagnosis and treatment of HCV infection, this is a great challenge for the medical professionals. To invite patients from excluded communities to participate in standard medical care is the key task that needs to be solved in order to achieve the WHO goal to eliminate HCV infection by 2030 [6].

## 2. Global Epidemic of Viral Hepatitis C

There is around 1% of the world’s population suffering chronic viral hepatitis C, declared by the recent WHO estimate. It means that approximately 71 million subjects exist with this serious chronic illness [1,7]. Viral hepatitis C is the leading cause of liver-related death. End-stage liver disease and consequences of chronic HCV infection lead to more than 670,000 deaths annually [8]. The HCV pandemic is unevenly distributed around the whole world although there are patients on all continents. There is a geographic diversity in the distribution of HCV genotypes globally. The leading causes of new HCV infections are also varied in different regions. In developed countries, the new cases are associated with injecting drug use, but in the developing countries, unsafe health-care procedures are still the problem and the main source of HCV transmission.

Global incidence of HCV infection, based on mathematical modelling, is estimated to be about 1.75 million new infections in 2015 [1]. The eastern Mediterranean and European regions, with the incidence of 62.5/100,000 and 61.8/100,000 population, respectively, are the most affected [1]. There are variations in the prevalence both across and within countries.

PWID have been identified as a major risk group for HCV transmission [2,4,9,10], which is attributable to needle and syringe sharing.

New outbreaks of HCV infection take place among people who use actively illicit drugs as well as among patients on opioid substitution therapy (OST). In the real world, these clients may continue to inject drugs in some (especially low-threshold) OST programs. According to the latest numbers from 2019, 6.1 million (8.6%) of 71 million HCV infected are PWID [11,12]. Globally, there is approximately 40% prevalence of viral hepatitis C among PWID [4,13]. Two-thirds of new VHC cases in Europe are related to injecting drug use [14].

## 3. WHO Global Elimination Plan

WHO presented an action plan to bring the viral hepatitis C pandemic under control in May 2016, describing the vision of global HCV infection elimination [4]. The Global Health Sector Strategy (GHSS) calls for the elimination of viral hepatitis as a public health threat by 2030.

**Figure 1:**
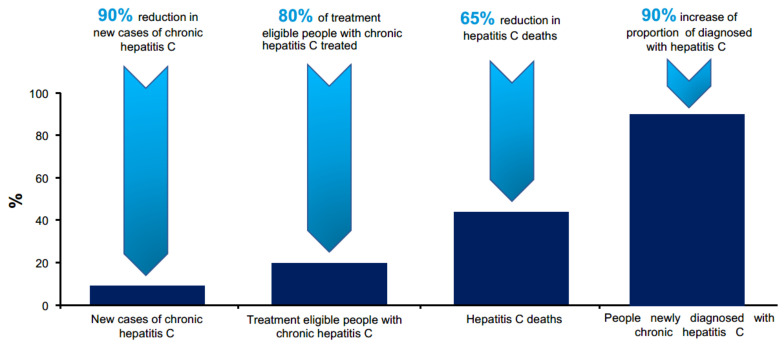
WHO goals in treatment of hepatitis C infection [4].

The elimination targets by WHO are defined as a 90% reduction of new infections and 65% reduction in mortality by 2030 compared with the 2015 baseline (Figure 1). To accomplish these two goals, a third step is necessary: to increase the proportion of diagnosed people with HCV infection up to 90% [4]. This is to be achieved through a combination of preventing transmission by improving blood safety and infection control measures, extending harm-reduction services aimed at reducing transmission among PWID, and expanding testing and DAA treatment for those already infected [1,4,15].

**Figure 2:**
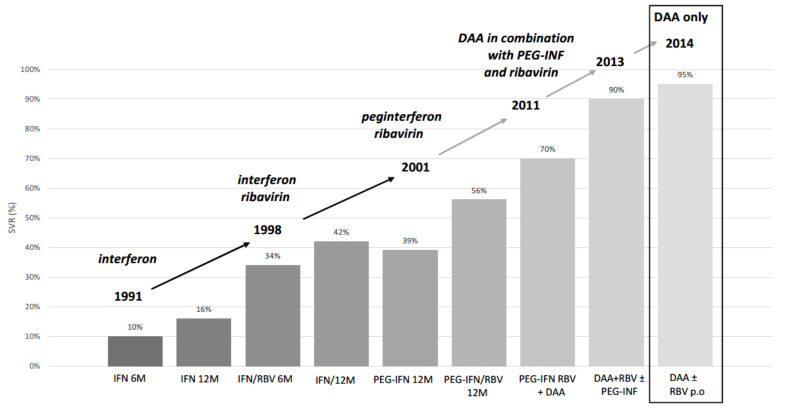
Evolution of chronic viral hepatitis C treatment modalities and increase in the efficacy of viral clearance measured as sustained virologic response [16]. SVR, sustained virological response; IFN, interferon; RBV, ribavirin; PEG-IFN, peginterferon (pegylated interferon); DAA, direct-acting antivirals.

The reduction in the number of HCV-related deaths (the first WHO goal) has become manageable and reachable since 2014. In this memorable year, the DAA regimens in therapy of chronic viral hepatitis C were introduced, and we have been able to cure this infection more efficiently since then (Figure 2).

**Figure 3:**
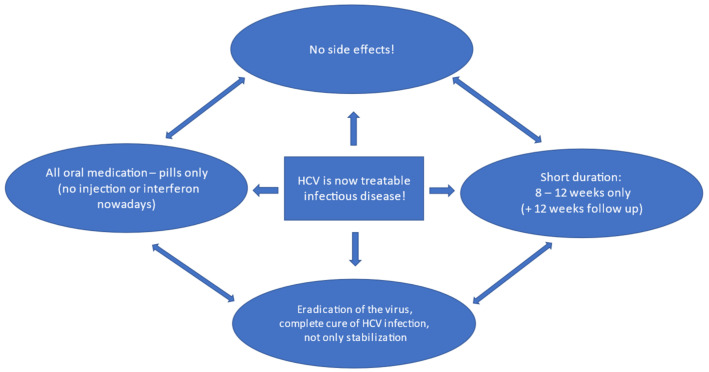
Viral hepatitis C treatment with DAA—key information to share with professionals from other fields as well as with potential patients.

The treatment with new all-oral DAA combinations is highly effective and safe even for the patients with viral hepatitis C-related advanced liver disease and cirrhosis (Figure 3). More than 80% of hepatocellular carcinoma cases are due to HCV infection [8,17], which is nowadays preventable with adequate therapy.

To decrease the incidence of HCV infection and possibly eliminate this infectious disease, we need to decrease the sources of infection outbreaks. Infected people are the source, and according to the modelling study, there is a need to treat and cure more than 10% of the infected population in each country to significantly decrease the risk of transmissions. However, only three countries (Germany, France, and the Netherlands) met this demanding request in the year 2015 [15].

According to the 2014 and 2016 updated WHO guidelines [2], less than 1% of people with chronic hepatitis infection were receiving treatment. The WHO strategy called for 3 million people with chronic hepatitis C virus to be treated by 2020. By 2030, treatment coverage for both chronic viral hepatitis B and C infection should reach 80% of eligible persons.

The most vulnerable group for frequent HCV transmission is undoubtedly PWIDs. The wide and intense spread of HCV infection can be controlled with a broad implementation of harm-reduction programs and unlimited DAA treatment in this subpopulation. The concept of treatment as prevention strategy is efficient among PWID [18].

To conclude, open access to diagnostic tools and unlimited accessibility of the new DAA therapy for all infected subjects are the keys to HCV elimination. It is also extremely important to treat them within a limited time frame of a few years. This approach is the only way to decrease transmission and reinfections among PWID as well as the selection of resistant HCV strains. There is an increasing discussion about the emergence of viral resistance to DAAs, especially to NS5A inhibitors. This could be a significant obstacle even if viral failures represent only a minor proportion of the DAA-treated patients [19,20,21].

In the next years, with the planned increase of treatment uptake, a large number of people may acquire resistant HCV globally. According to current estimate, the virological failures can range from 2% [22] to 5–10% [23,24] of patients, respectively. The circulation of resistant strains (novel, induced after unsuccessful DAA treatment, or mixed) calls for continuous surveillance to develop the most effective strategies for viral hepatitis C management worldwide [21]. The transmission of DAA-resistant HCV strains may affect local cure rates and thus may jeopardize global eradication efforts [25]. Therefore, it is necessary to take action immediately. If we miss the proper time, we may lose the opportunity to eliminate HCV infection because the viral resistance could threaten global elimination.

## 4. Process of Viral Hepatitis C Diagnosis

**Figure 4:**
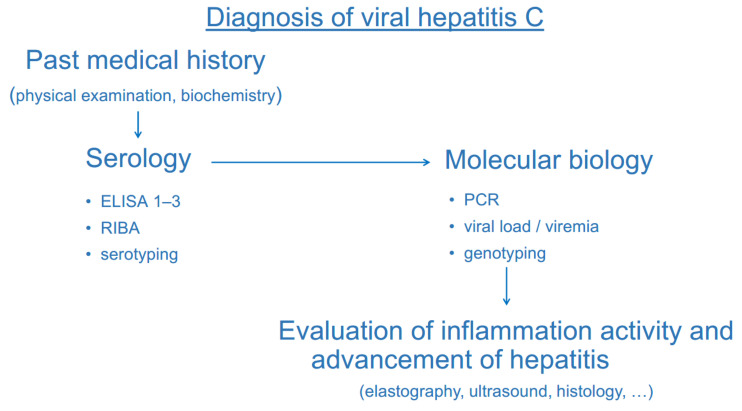
Diagnostic procedures of viral hepatitis C.

Due to the lack of clear clinical picture and physical findings on examination, the diagnosis of viral hepatitis C is based on specific serologic and virologic laboratory tests. When obtaining past medical history from a suspected patient, we must think of viral hepatitis C and ask questions about risk factors, such as injecting drug use, imprisonment, or tattooing. Elevation of liver function tests is neither always present nor specific, and it is not diagnostic for viral hepatitis C. With any kind of recent or past risk behaviour, specific viral hepatitis C testing is necessary (Figure 4).

The first step is the anti-HCV antibody test by ELISA (enzyme linked immunosorbent assays) or EIA (enzyme immunoassay). Antibody tests show a false-negative result early after the infection in the acute phase of this viral disease. There is a so-called “diagnostic window”, when the antibody tests become positive 6–12 weeks after the infection depending on the sensitivity of a particular test method.

The latest tests use the combination of antigen-antibody assays. Two markers of the same infection are detected at the same time with average window period of only 26.8 days [26].

At the same time, during the acute phase of HCV infection, HCV RNA replicates most rapidly, and the patient is highly infectious due to the high viral load in their blood [27].

The diagnosis of viral hepatitis C is based on anti-HCV antibody detection followed by the confirmation of viral RNA by a polymerase chain reaction (PCR) test.

PCR-based techniques are widely used for diagnosis and confirmation of HCV infection. The viral load is measured in IU/mL [28]. These days, the PCR testing is usually performed with fully automatic, closed PCR amplifying systems. At the same time, we can also measure the presence of the virus in a blood sample (positive × negative) as well as the viral load by quantitative assessment [29,30,31]. The standardised tests are very sensitive, with the lower limit < 15 IU/mL.

PCR-based techniques are also available to genotype or subtype HCV [29,30,31]. These detailed tests used to be important before the treatment of an infected patient. At least six major HCV genotypes are recognized with varying characteristics, one of which is different susceptibility to treatment. Nowadays, they are not of critical importance due to the broadly active, pangenotypic treatment regimens (see more in the section on treatment).

To access the progression of the disease and to evaluate the liver tissue damage, imaging methods such as ultrasound, CT scan, and MRI are usually used.

In some countries, however, biochemical assays are utilized to evaluate the liver tissue damage. FIB-4 index measures fibrosis from the values of patients age, platelet count, aspartate aminotransferase (AST), and alanine aminotransferase (ALT), while APRI (AST to platelet ratio) index predicts fibrosis and cirrhosis according to a AST platelet count only.

In the past, the “gold standard” for evaluating the liver pathology was the liver biopsy with which the levels of fibrosis or cirrhosis as well as the intensity of inflammation were assessed.

The level of fibrosis is an important prognostic indicator. It influences not only the decision of how to treat the given patient but also the choice of follow-up protocols and screening programs [28].

Currently, the liver biopsy has been replaced by a non-invasive technique: elastography. This new technology gives us information about the stiffness of liver tissue and distinguishes advanced fibrosis and cirrhosis without any pain and risk of complications. The most common is VCTE (vibration-controlled transient elastography). Another technology is SWE (shear-wave elastography) based on ultrasound. For each liver disease (viral hepatitis C, viral hepatitis B, alcoholic liver disease, non-alcoholic fatty liver disease (NAFLD), etc.), there are different stiffness limits, which correlate with classification of liver fibrosis, according to the METAVIR score system (F1–4) [32]. There are many advantages of elastography: it is easy to perform, and examination is not invasive, and that is why elastography can be performed also on an outpatient basis even outside of a medical facility. As already mentioned, it is easy to perform and can be operated by certified nurses or X-ray technicians rather than by medical doctors. In the current world, this is very important: effortlessness of this diagnostic technique and unambiguous result (number) increases the accessibility and thus helps to speed up the diagnosis and to shorten the time between the engagement and the beginning of treatment in fieldwork.

## 5. Viral Hepatitis C Diagnosis in Present Practice

It appears complicated, but the entire diagnostic process can be done within one visit. The most important thing is to keep this disease in mind when working with PWID. When solving the problems related to the addiction to psychoactive substances, everyone who works with PWID should think of the most common infectious diseases related to the injecting drug-use practice. Here, we must emphasize the important role of potentially collaborating specialists: addictologists, social workers, and other non-medical professionals who regularly communicate with PWID. They can easily provide PWID with information about infection risks as well as the necessity of knowing their own infectious status. The knowledge of their health status is the most important first step towards a PWID‘s long way to recovery.

The role of field workers and addictologists nowadays is crucial. They may serve as a bridge between PWID and medical services. In the treatment of chronic HCV infection, “the peer to peer model” is not efficient enough, so addictologists must take the opportunity and offer their help in communicating, escorting PWID to the medical floors in hospitals, and also connecting them with the health care system. We all know that besides some improvements in the last few years, the treatment uptake of patients from excluded sub-populations (PWID, homeless, or prisoners) is insufficient so far [6].

As we mentioned earlier, testing for viral hepatitis C is easy. This is also a very important piece of information for PWID. One of the barriers to viral hepatitis C diagnostics and therapy is that both the PWID and also some professionals, who are not exactly focused on viral hepatitis, may have slightly limited and not updated knowledge of diagnostic procedures and viral hepatitis C treatment options.

According to common public opinion, people still believe that the diagnostic process is time consuming and complicated and that there is no treatment for chronic viral hepatitis C. This is a major misconception that has to be corrected for the success of the WHO‘s elimination plan. The key message of this article is that we, hepatologists and infectious disease specialists, have to communicate the new knowledge to the professionals who take care of PWID. We hope that the new knowledge will, in this way, spread soon inside the PWID community.

In health care facilities used to working with PWID, the diagnostic process involves just one simple blood draw, done in one day. The complete results are usually ready in a few days. On the second visit to the doctor‘s office, the results are discussed, and fibroscan or ultrasound scanning can be performed. Thus, the diagnostic tools are completed: easy, quick, and painless.

Once diagnosed with chronic HCV infection, PWID are eligible for specific virostatic treatment according to international guidelines as well as WHO recommendation.

## 6. Viral Hepatitis C Treatment with Direct Acting Antivirals

Quite a remarkable revolution has recently begun in the field of viral hepatitis C therapy [33]. The treatment of viral hepatitis C with DAA began in 2014. The new antiviral medications are targeted to viral enzymes and proteins only, have good tolerance profiles and excellent efficacy, and offer a unique opportunity to achieve HCV infection elimination worldwide. The primary goal of treatment is to achieve sustained virological response (SVR), defined as undetectable serum HCV RNA 12 weeks after the end of treatment [34]. The SVR indicates that viral infection has been cured [35,36].

Eradication of HCV infection is associated with regression of fibrosis and liver cirrhosis, improvement in clinical outcome, and survival [37]. It is also associated with a decreased incidence of complications, such as end-stage liver disease or hepatocellular carcinoma (HCC). The benefit of the survival of DAA-treated subjects with decompensated cirrhosis was recently also confirmed [38].

Interferon-free DAA combinations can nowadays cure viral hepatitis C in more than 95% of patients with HCV infection. This new treatment is not only more efficient but also significantly shorter, with an average duration of 8–12 weeks. Three major classes of drugs with different modes of action are available: NS3 protease inhibitors, NS5A inhibitors, and NS5B inhibitors. Currently, fixed-combination regimens combining two or three different classes of antivirals in one pill are available. Some fixed combinations have pangenotypic efficacy, which makes genotyping less important. Unlike in the U.S., in some countries (for example, in the Czech Republic), genotyping is still requested before treatment as a cornerstone of the diagnostic process.

The treatment possibilities in certain clinical situations are summarised in Table 1 to give an overview of the options we have nowadays. The updated, simplified version not requiring genotyping before the treatment is shown in Table 2.

## 7. Viral Hepatitis C Treatment with Direct-Acting Antivirals

Currently, the HCV-infected PWID patients themselves represent the major obstacle to global HCV infection eradication. PWID patients are usually considered unreliable, incapable of keeping medical appointments, and above all, due to their addiction to illicit drugs, they behave differently from the other patients. Honestly, there are several underlying reasons that lead to the chronic problem of PWID non-compliance: one of them is that the health care system is usually designed not for PWID but for the so-called “common population”.

This mismatch is reciprocal. Outside of specialized addictology services and psychiatry, there is rarely anyone voluntarily willing to work with PWID. Based on practice, unequivocally, they are the most difficult patients for the majority of all health care professionals: the doctors, nurses, as well as the medical staff.

There are more causes of this state of antipathy, and some of them are rather emotional than rational.

**Figure 5:**
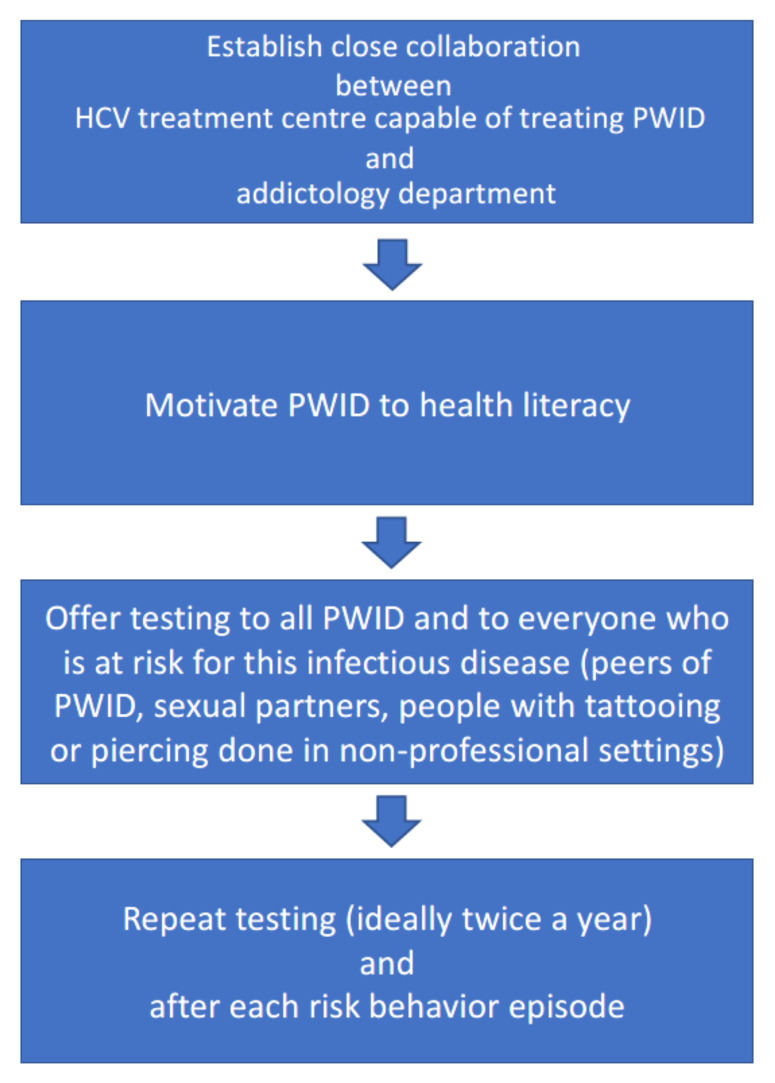
Viral hepatitis C diagnosis—simplified summary.

The majority of PWID are socially marginalized due to their behavior under the illicit drugs or even psychiatric disease and comorbidity, low income, poverty, homelessness, as well as incarceration in past, etc. Unfortunately, for all these reasons, the PWID themselves limit their access to health care services. They also suffer from a lack of adequate information. Education of PWID is the way to solve this problem, but it is extremely difficult to accomplish. The most efficient way is education by fieldworkers and harm-reduction programs. Due to the stigma of being PWID, they have difficulties in visiting health care facilities others than those specifically designed for them (Figure 5).

In general, medical professionals (other than addictologists) are often not able to communicate with PWID properly, partly because they are subliminally in conflict with PWID and with their self-destructive behaviour. They also lack the ability to motivate the PWID into better cooperation with them first because they have, again, their own internal conflict, and second, they are not willing to collaborate with PWID. This needs to be changed. For these medical professionals not used to having contact with PWID, the close cooperation with addictologists could be of great help and may bypass the problematic communication and connection with PWID patients with somatic diseases. It is time to go beyond our standard routine algorithms and approaches and prepare to collaborate with others to address PWID patients properly.

## 8. Conclusions: Global Elimination—Our Common Road to Our Common Goal

In theory, we have everything available to achieve successful elimination of HCV infection in accord with the WHO plan:

- Diagnostic tools are currently sensitive and specific enough to diagnose HCV infection in all infected subjects;
- Safe and accurate techniques to measure the severity of liver tissue damage, eliminating painful liver biopsy;
- “All-oral” treatment with the new DAA for chronic viral hepatitis C is available. The new antiviral medications have good tolerance profiles and excellent efficacy, thus offering a unique opportunity to achieve HCV infection elimination worldwide.

These tools and resources are ready to be used by skilled professionals: hepatologists as well as infectious disease specialists are both well-trained in treating viral hepatitis C.

**Figure 6:**
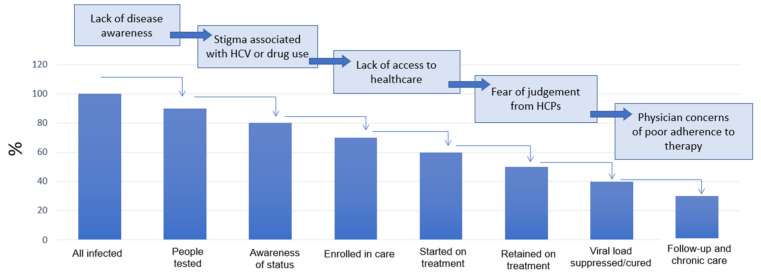
WHO treatment cascade [1]. HCPs—healthcare professionals.

As already mentioned, in theory, we have everything available, but in practice, there is one unsolved problem: how to get the marginalized patients into doctors’ offices (Figure 6).

Addictologists know how to communicate with PWID and motivate them to change their lifestyle. To succeed in HCV infection eradication, these two professional groups should get together and collaborate closely. Only the triple combination (PWID–addictology–medicine) can lead to success of global HCV infection elimination.

Open access to diagnostic tools and unlimited accessibility of therapy for all infected subjects are the two well-known steps to HCV elimination. Establishing contact between PWID and medical doctors who are co-operative and willing to treat and cure infected PWID is the springboard to global elimination of HCV infection. It is difficult, time consuming, and exhausting, but without the interdisciplinary cooperation in creating this connection, we cannot expect any quantitative change, and only few PWID will be diagnosed and cured, as in present practices. These multi-disciplinary and collaborative efforts are the crucial steps for effective HCV infection elimination [23,24]. They are at least as important as the newest, most sensitive PCR tests for diagnostic or the latest, third-line rescue DAA therapy.

Adequate involvement of high-risk sub-populations in the treatment cascade is important if not the most important task, and that is why the elimination of HCV infection represents such a challenge.

## Figures and Tables

**Table 1:** Treatment modalities for patients without liver cirrhosis [39].

Patients Genotype	Prior Treatment Experiance	SOF/VEL	GLE/PIB	SOF/VEL/VOX	SOF/LDV	GZR/EBR	OBV/PTV/r + DSV
Genotype 1a	Treatment-naive	12 wk	8 wk	No	8–12 wk	12 wk (HCV RNA ≤800,000 IU/mL)	No
	Treatment-experienced	12 wk	8 wk	No	No	12 wk (HCV RNA ≤800,000 IU/mL)	No
Genotype 1b	Treatment-naive	12 wk	8 wk	No	8–12 wk	8 wk (F0–F2)12 wk (F3)	8 wk (F0–F2)12 wk (F3)
	Treatment-experienced	12 wk	8 wk	No	12 wk	12 wk	12 wk
Genotype 2	Treatment-naive	12 wk	8 wk	No	No	No	No
	Treatment-experienced	12 wk	8 wk	No	No	No	No
Genotype 3	Treatment-naive	12 wk	8 wk	No	No	No	No
	Treatment-experienced	12 wk	12 wk	No	No	No	No
Genotype 4	Treatment-naive	12 wk	8 wk	No	12 wk	12 wk (HCV RNA ≤800,000 IU/mL)	No
	Treatment-experienced	12 wk	8 wk	No	No	No	No
Genotype 5	Treatment-naive	12 wk	8 wk	No	12 wk	No	No
	Treatment-experienced	12 wk	8 wk	No	No	No	No
Genotype 6	Treatment-naive	12 wk	8 wk	No	12 wk	No	No
	Treatment-experienced	12 wk	8 wk	No	No	No	No

DSV, dasabuvir; EBR, elbasvir; GLE, glecaprevir; GZR—grazoprevir; LDV—ledipasvir; OBV—ombitasvir, PIB—pibrentasvir; PTV—paritaprevir; r—ritonavir; SOF—sofosbuvir; VEL—velpatasvir; VOX—voxilaprevir.

**Table 2:** Simplified treatment modalities for patients without or with compensated liver cirrhosis [40].

Type of Treatment	Patients Genotype	Prior Treatment Experience	Cirrhosis Status	SOF/VEL	GLE/PIB	SOF/VEL /VOX	GZP/EBR
Simplified treatment no genotype/subtype determination	All genotypes	Treatment-naive	no cirhosis	12 wk	8 wk	NO	NO
		Treatment-experienced					
		Treatment-naive	compensated (Child-Pugh A)		12 wk		
		Treatment-experienced					

DSV—dasabuvir; EBR—elbasvir; GLE—glecaprevir; GZR—grazoprevir; LDV—ledipasvir; PIB—pibrentasvir; SOF—sofosbuvir; VEL—velpatasvir; VOX—voxilaprevir.
